# The big and small ball sign: Ultraviolet-Induced Fluorescence Dermoscopy for the diagnosis of scabies

**DOI:** 10.1590/0037-8682-0238-2024

**Published:** 2025-01-27

**Authors:** Kaique Arriel, Thaís Barros Felippe Jabour, Rafael Rubinho, Thais Kohatsu Yanase, Fernanda Rytenband

**Affiliations:** 1 Universidade de Santo Amaro, Departamento de Dermatologia, São Paulo, SP, Brasil.

**FIGURE 1:**
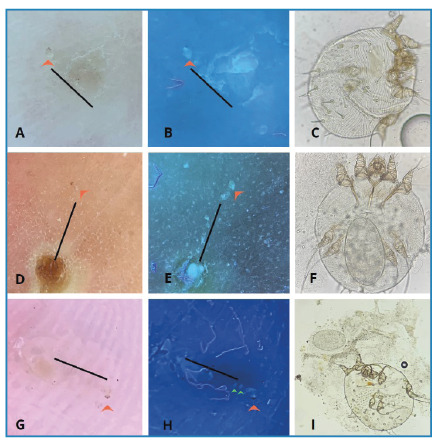
In panels A, D, and G, dermoscopy with polarized light shows tunnels outlined by black lines and the delta glider sign indicated by red arrows. Panels B, E, and H display the tunnel and the mite's body in a greenish-white color under UV light dermoscopy, referred to as the "big ball sign." Panels C, F, and I depict *Sarcoptes scabiei* under direct optical microscopy. In panel H, two smaller oval structures appear in a greenish-white color, marked by green arrows, representing the eggs ("small ball sign"). In panel C, an egg is visible next to the mite, and in panel I, an egg is shown inside the mite.

This paper discusses a case involving a family of four who presented with a three-month history of persistent itching. Examination revealed diffusely distributed excoriated erythematous papules. Dermoscopy with polarized light made it difficult to identify tunneling and the delta glider sign in the lesions. In contrast, dermoscopy with UV light (DL5, light 365nm, Dermlite®) successfully identified the tunnels and exhibited the delta glider sign at their ends, in addition to revealing both larger and smaller oval structures in a greenish-white color (Figure 1). Superficial curettage using a scalpel blade was performed at the site of these findings until the features were no longer visible. The collected material was then placed on a slide with 10% potassium hydroxide and examined under optical microscopy, confirming the presence of scabies mites and eggs. 

Dermoscopy demonstrates up to 91% sensitivity in diagnosing scabies and yields superior results when combined with skin curettage. Recent studies indicate that dermoscopy with UV light facilitates rapid identification of the tunnels and mites, showing an oval greenish-white reflection known as the "ball sign”, as described by Yürekli^1^ ^,^ ^2^. This study presents what is believed to be the first identification of eggs left by the mite within the tunnels using UV light dermoscopy. These eggs are greenish-white, oval structures approximately 25% the size of the mite. Expanding on Yürekli's description, we introduce the "big and small ball sign" to describe the images of *Sarcoptes scabiei* mites and their eggs observed through UV light dermoscopy.
